# Supplementary material for: Relationship between serum bilirubin levels, urinary biopyrrin levels, and retinopathy in patients with diabetes
Source: PLoS One. 2021 Feb 11;16(2):e0243407. doi: 10.1371/journal.pone.0243407 (PMC7877782; doi:10.1371/journal.pone.0243407)
Supplement: S1 File — (PDF) [file pone.0243407.s001.pdf]

Patient Nu	DBP	SBP	Sex(men, 1)	Cr	Age	Age/10	BMI	Duraion
1	76	150	1	0.35	64	6.4	19.72	1
2	73	118	0	0.39	15	1.5	24.46	1
3	88	157	0	0.35	59	5.9	31.18	1
4	85	137	1	0.59	25	2.5	30.12	10
5	78	115	1	0.47	65	6.5	21.05	10
6	76	108	0	0.43	34	3.4	45.52	2
7	88	136	1	0.56	36	3.6	25.68	12
8	87	164	1	0.48	66	6.6	24.01	
9	80	140	0	0.39	58	5.8	31.56	10
10	85	163	0	0.39	60	6	32.39	25
11	79	122	0	0.5	27	2.7	39.35	6
12	78	122	1	0.55	58	5.8	22.23	1
13	67	113	0	0.4	72	7.2	20.2	3
14	80	131	0	0.47	40	4	17.44	11
15	88	145	0	0.45	51	5.1	24.56	2
16	70	108	0	0.45	51	5.1	25.96	4
17	74	142	0	0.44	57	5.7	28.16	1
18	100	176	0	0.45	53	5.3	27.39	0.1
19	69	117	0	0.44	61	6.1	28.67	20
20	88	123	1	0.66	43	4.3	30.48	1
21	69	112	1	0.73	33	3.3	18.71	0.5
22	69	105	1	0.68	44	4.4	25.86	9
23	71	111	0	0.48	58	5.8	26.71	0.1
24	93	180	1	0.63	63	6.3	31.1	14
25	87	140	0	0.49	63	6.3	17.8	0.05
26	79	127	0	0.5	60	6	29.67	3
27	84	123	0	0.51	57	5.7	32.88	1
28	70	113	1	0.64	70	7	24.61	9
29	85	156	0	0.48	75	7.5	26.12	6
30	66	99	0	0.56	42	4.2	16.33	0.5
31	83	141	0	0.48	78	7.8	24.65	4
32	87	125	0	0.56	44	4.4	28.28	17
33	73	107	1	0.73	47	4.7	26.35	10
34	75	124	0	0.49	78	7.8	20.93	0.05
35	79	159	1	0.65	82	8.2	24.22	8
36	90	128	1	0.78	41	4.1	29.07	4
37	84	162	1	0.72	59	5.9	26.49	7
38	92	144	0	0.59	44	4.4	28.72	3
39	88	125	1	0.77	46	4.6	24.62	0.1
40	77	150	1	0.74	55	5.5	24.86	20
41	64	137	1	0.67	81	8.1	17.86	0.1
42	73	119	1	0.74	56	5.6	29.03	2
43	81	121	1	0.81	41	4.1	23.44	2
44	64	116	1	0.74	59	5.9	22.68	3
45	76	132	1	0.72	67	6.7	28.48	15
46	74	121	0	0.55	66	6.6	30.18	4
47	84	120	1	0.8	47	4.7	22.53	
48	99	154	1	0.77	63	6.3	23.15	10
49	66	116	1	0.79	62	6.2	25.82	21
50	66	127	0	0.58	76	7.6	19.31	3
51	66	106	1	0.81	62	6.2	16.9	5
52	66	122	0	0.59	75	7.5	25.15	17
53	68	98	1	0.85	56	5.6	15.43	1
54	70	112	0	0.64	59	5.9	22.77	7
55	81	153	0	0.63	64	6.4	29.94	10
56	78	118	0	0.64	61	6.1	19.82	12
57	73	117	1	0.97	36	3.6	24.44	
58	79	129	0	0.63	68	6.8	22.03	8

59	80	130	1	1.01	33	3.3	21.8	4
60	47	97	0	0.75	37	3.7	19.56	14
61	84	161	0	0.66	65	6.5	20.55	39
62	87	148	0	0.69	56	5.6	27.7	7
63	65	116	1	0.86	70	7	19.95	30
64	82	124	1	0.87	67	6.7	24.86	20
65	72	109	1	0.88	65	6.5	20.2	
66	75	117	1	0.9	61	6.1	24.51	2
67	69	154	0	0.64	79	7.9	23.93	3
68	78	168	1	0.85	79	7.9	22.83	24
69	84	138	1	0.89	68	6.8	23.39	5
70	76	141	1	0.9	66	6.6	24.86	
71	87	154	1	0.87	76	7.6	23.14	14
72	62	111	1	0.91	65	6.5	20.44	3
73	103	177	1	1.01	45	4.5	39.89	1
74	66	119	1	0.9	70	7	26.4	40
75	76	110	1	0.96	55	5.5	23.46	
76	83	173	1	0.86	85	8.5	19.88	0.3
77	49	110	1	0.95	69	6.9	23.15	
78	75	122	1	0.98	72	7.2	17.65	20
79	92	192	0	0.8	82	8.2	24	7
80	83	145	0	0.87	62	6.2	22.6	31
81	68	138	1	1.12	70	7	27.18	30
82	76	141	1	1.24	70	7	24.16	32
83	68	107	0	1.26	28	2.8	31.62	12
84	98	198	1	1.41	54	5.4	24.24	21
85	64	96	0	1.12	66	6.6	20.34	10
86	68	158	1	1.46	79	7.9	19.43	29
87	82	167	0	1.22	73	7.3	25.11	21
88	116	167	1	2.21	32	3.2	27.1	18
89	110	164	1	2.63	63	6.3	23.53	31
90	62	133	0	2.01	81	8.1	29.82	13
91	106	195	0	2.95	66	6.6	24.52	7
92	88	163	1	4.35	59	5.9	21.89	12
93	77	185	1	5.02	69	6.9	21.3	20
94	99	175	1	8.97	34	3.4	23.46	

smoking	HbA1c	FBG	T.Bil	10×T. Bil	D.Bil	LDL	TG	HDL	eGFR
1	8.5	117	1.1	11	0.4	48	37	29	185.4461
0	13.8	178	0.7	7	0.1	168	242	43	184.6219
0	11	220	0.8	8	0.1	164	155	55	140.2818
0	9	143	1.2	12	0.3	135	147	33	137.1769
0	9.9	154	0.7	7	0.2	54	73	58	133.7274
0	8.4	173	0.3	3	0.1	97	116	50	131.1889
0	8.2	107	0.7	7	0.1	57	154	32	130.8053
1	12.7	349	0.5	5		166	211	40	130.1112
0	9.2	183	0.7	7	0.2	144	56	65	125.2327
0	8.9	202	0.9	9	0.2	167	61	81	124.0201
0	9.5	247	1.4	14	0.4	107	243	38	118.8424
1	9.2	137	1.1	11	0.4	205	385	62	116.3432
0	9.6	140	0.6	6		67	70	48	114.4822
0	13	252	0.5	5	0.1	122	52	63	113.6004
0	7.2	137	1.1	11		86	131	39	111.1115
1	14.8	222	0.6	6	0.1	144	172	40	111.1115
1	12.2	176	0.8	8		120	171	46	110.2993
0	11.1	115	0.9	9	0.1	158	133	43	109.8916
0	11.5	224	0.5	5	0.1	141	139	46	108.1731
0	8.1	139	0.9	9	0.2	93	240	40	103.8521
0	12.8	230	1.1	11	0.3	119	108	71	100.349
1	8.1	99	0.5	5	0.1	124	87	64	99.85414
1	13.9	224	0.8	8	0.2	143	74	44	99.78481
0	8.2	115	1	10	0.2	125	33	100	97.92925
0	6.8	90	0.6	6	0.1	87	96	74	95.27104
0	8.8	211	0.9	9	0.2	115	171	46	94.50256
0	10.9	164	0.9	9	0.2	113	82	51	93.84871
1	10.2	154	1.1	11	0.3	104	51	32	93.38942
0	9.6	85	0.7	7	0.1	198	139	92	92.68846
0	10.3	279	1	10	0.2	71	153	78	92.48172
0	9.2139	139	0.9	9		82	67		91.65098
1	10.8	269	0.6	6	0.1	108	519	37	91.25518
1	11.8	154	0.8	8	0.2	53	140	65	90.66391
1	7.3	171	0.5	5	0.1	110	137	46	89.6067
1	10.6	205	1	10	0.2	108	93	41	87.74249
0	11	200	0.6	6	0.1	152	180	27	87.6963
1	9.8	184	0.8	8	0.1	120	567	38	86.22743
0	8.4	185	0.3	3		113	95	43	86.19124
0	11.9	238	0.8	8	0.2	122	103	46	86.05367
1	11.1	184	0.6	6	0.2	53	125	31	85.38432
0	7.4	254	0.9	9	0.2	108	154	49	85.1806
1	12.1	235	0.7	7	0.2	111	135	47	84.94391
0	6.7	67	0.3	3	0.1	84	101	33	84.14923
1	11.8	153	0.9	9	0.3	39	52	52	83.68116
1	9.5	135	0.9	9	0.2	106	158	31	83.13741
1	7.4	145	0.6	6	0.1		174	38	82.84767
1	7.2	110	0.6	6		110	79	35	82.02178
1	8.2	159	0.8	8	0.1	153	283	41	78.62672
1	6.9	106	0.5	5	0.1	56	33	73	76.80355
0	10.9	118	1.1	11	0.3	89	117	44	75.06931
1	7.8	225	0.7	7	0.2	64	47	62	74.73133
0	12.2	197	1	10	0.2	167	179	63	73.95908
1	8.1	144	0.4	4	0.1	175	238	42	72.99405
0	11.4	92	0.8	8		98	134	47	72.48543
0	11.5	140	0.8	8	0.1	148	123	54	72.04336
0	10.1	107	0.7	7	0.1	114	104	35	71.79523
1	12	246	0.6	6	0.1	152	355	34	71.71576
0	9	51	1.7	17	0.3	145	51	59	70.8007

1	8.4	82	1.2	12	0.3	151	77	62	70.34944
1	8.4	261	1	10	0.5	60	45	35	69.67151
0	7.7	111	0.7	7	0.1	89	66	79	68.16461
1	7.8	130	0.8	8	0.2	82	193	47	67.76654
0	9	187	1.2	12	0.3	99	37	46	67.59542
1	11.8	187	0.6	6	0.2	105	105	39	67.59027
1	6.8	130	0.3	3	0.1	63	99	59	67.33356
1	7.9	114	0.8	8	0.2	92	143	40	66.90688
0	7.2	156	0.8	8	0.2	84	73	54	66.66015
0	6.6	103	0.8	8	0.2	151	90	38	66.12999
1	10.2	216	1	10	0.2	147	166	34	65.65065
1	10.2	142	0.8	8	0.2	54	106	37	65.41108
1	9	150	0.8	8	0.1	164	239	53	65.18898
1	9.6	168	0.9	9	0.2	102	60	61	64.90891
0	8.7	255	0.4	4	0.1	175	327	39	64.35794
0	7.2	115	0.7	7	0.2	55	105	55	64.31574
1	10.2	173	1	10	0.2	185	227	49	64.2263
1	9.3	86	0.6	6	0.1	93	112	48	63.93184
1	6.9	169	0.3	3	0.1	84	169	23	60.87268
0	7.9	151	0.4	4	0.1	89	117	37	58.12273
0	6.7	102	0.8	8	0.1	158	117	47	51.66556
1	7.6	115	0.7	7	0.1	151	126	39	51.07351
1	11.6	357	0.6	6	0.1	161	154	34	50.63072
1	8.5	155	0.3	3	0.1	66	80	36	45.29552
1	9.7	188	0.3	3	0.1	161	110	42	42.78647
0	7.2	155	0.9	9		336	268	53	42.39933
0	7.4	94	0.6	6	0.1	96	89	61	38.05332
1	6.3	104	0.8	8	0.1	132	141	43	36.59154
1	8.4	101	0.6	6		121	149	40	33.66626
0	7.5	216	0.2	2	0.1	231	75	52	30.13412
0	7.4	65	0.6	6	0.1	108	585	34	20.5097
1	7.8	156	0.9	9	0.3	101	193	27	18.92407
1	8.2	158	0.3	3		120	267	61	13.19021
1	6	90	0.3	3	0.1	105	261	32	12.05202
1	7.6	114	0.5	5	0.1	104	150	40	9.851042
1	5.9	87	0.2	2	0.1	103	67	41	6.396057

eGFR ≤30	hsCRP	Biopyrrin	HO-1	dROM	retinopath	SDR	PDR
0	189	5.487724839	7.21	200	0	0	0
0	394	2.08183621	3.04	278	0	0	0
0	1240	0.688277638	5.36	552	1	0	1
0	719	1.88689641	6.78	265	0	0	0
0	3280	11.39632048	6.85	437	1	0	0
0	14800	3.454928673	6.65	653	0	0	0
0	1360	3.81143333	4.09	275	1	0	1
0	1430	1.615063686	7.16	366	0	0	0
0	773	2.333316202	3.95	254	1	0	0
0	388	0.410032052	3.08	296	1	0	1
0	329	6.176828393	11.58	262	0	0	0
0	1350	13.57052246	7.8	365	0	0	0
0	23900	2.343727234	15.38	184	0	0	0
0	925	0.528729907	2.4	397	1	0	1
0	621	2.414062409	9.32	289	0	0	0
0	357	1.188027952	3.04	331	0	0	0
0	2290	1.067834567	6.87	307	0	0	0
0	951	2.084531609	4.74	313	1	0	1
0	1390	3.89147376	5.27	413	1	0	0
0	1340	4.951978443	2.71	377	0	0	0
0	96	2.574205008	7.96	231	0	0	0
0	218	0.910199034	2.83	522	1	0	1
0	329	3.647799303	5.51	287	0	0	0
0	2520	1.566496374	5.07	306	1	0	1
0	590	0.428680328	3.63	258	0	0	0
0	3110	2.016848884	12.53	293	0	0	0
0	2970	2.562915142	8.15	274	1	1	0
0	64	1.813480512	5.46	289	0	0	0
0		1.028337017	10.91		1	0	0
0	309	0.588835346	8.15	435	0	0	0
0	1880	5.044465603	5.72	343	1	1	0
0	3330	3.541350567	3.58	361	1	1	0
0	758	3.648386483	7.85	292	1	0	0
0	370	1.337666084	5.33	219	0	0	0
0		1.727859026	6.6	279	0	0	0
0	4650	2.05592606	2.47	391	0	0	0
0	369	0.978526381	4.75	322	1	1	0
0	3270	0.817375066	4.03	385	0	0	0
0	356	1.348393296	7.23	259	0	0	0
0	169	1.107675021	2.82	367	1	1	0
0	293	0.861836785	9.89	263	0	0	0
0	751	0.437541215	9.12	319	0	0	0
0	1380	1.045693567	5.47	346	0	0	0
0	322	1.719466566	7.51	208	1	0	0
0	300	4.043264687	6.75	274	0	0	0
0	14100	1.462135641	7.35	383	0	0	0
0	516	1.183654683	5.38	226	1	0	1
0	159	1.221514842	4.18	265	1	1	0
0	118	1.213204916	6.98	251	0	0	0
0	121	1.237224374	3.85	204	0	0	0
0	563	5.190647177	10.09	320	1	1	0
0	532	1.835972305	6.68	334	1	1	0
0		0.576028507	4.84	470	0	0	0
0	1120	0.586196767	6.89	395	1	1	0
0	948	2.715914659	8.56	262	1	1	0
0	682	1.561066786	2.77	200	0	0	0
0	586	1.076064373	6.03	427	1	0	1
0	205	0.920169186	3.66	308	1	1	0

0	100	0.466693806	3.92	267	0	0	0
0	13700	13.48904435	5.13	295	0	0	0
0	436	0.37360487	5.36	244	1	0	1
0	246	2.225656315	3.3	279	1	0	1
0	160	0.86386688	3	262	0	0	0
0	416	0.620049551	8.04	290	1	0	0
0	4640	0.909964432	3.24	363	0	0	0
0	384	0.632274433	4.66	417	0	0	0
0	384	0.690346743	11.65	325	0	0	0
0	171	1.222218653	7.18	185	1	1	0
0	337	1.062551581	0.9	270	0	0	0
0	1540	1.201254149	8.37	319	1	1	0
0	961	0.626668782	8.76	206	0	0	0
0	1290	0.66961703	6	314	0	0	0
0	499	2.592902294	2.58	309	0	0	0
0	104	0.523717177	3.62	247	0	0	0
0	268	0.708174122	5.75	277	0	0	0
0	499	0.853427862	6.24	271	0	0	0
0		0.555310057	7.87	235	0	0	0
0		0.46012007	5.55	302	0	0	0
0	1310	0.934337193	6.63	248	1	1	0
0	621	0.762727579	3.5	377	1	0	1
0	6030	1.036958143	4.74	499	0	0	0
0	4160	0.643805103	10.05	365	1	0	1
0	235	0.20400723	1.75	267	1	0	0
0	387	2.768630169	6.57	261	1	0	1
0	723	0.661107207	8.47	493	0	0	0
0	1820	1.397782678	3.46	317	1	0	1
0	516	0.434749777	7.01	403	1	0	0
0	459	1.525615944	6.81	240	1	0	1
1	1220	0.663521264	6.41	250	1	0	1
1	3940	3.432812633	7.74	312	1	1	0
1		0.363472203	6.39	264	1	0	0
1	1870	1.191430673	5.95	338	1	0	0
1	684	0.584361997	2.87	254	1	0	1
1	447	0.866562154	5.08	348	1	0	1

PDR	PPDR	Nephropat	Nephropyl	Nephropth	Macroalb	Nephropthy +−
	0	0	0	0	0	0
	0	0	0	0	0	0
	1	0	0	0	0	0
	0	0	0	0	0	0
	1	0	0	0	0	0
	0	0	0	0	0	0
	1	1	0	0	0	1
	0	0	0	0	0	0
	1	0	0	0	0	0
	1	1	0	0	0	1
	0	0	0	0	0	1
	0	1	0	0	0	1
	0	0	0	0	0	0
	1	1	0	0	0	1
	0	0	0	0	0	0
	0	0	0	0	0	0
	0	0	0	0	0	0
	1	0	1	0	1	1
	1	0	0	0	0	0
	0	0	0	0	0	0
	0	0	0	0	0	0
	1	1	0	0	0	1
	0					
	1	1	0	0	0	1
	0	0	0	0	0	0
	0	0	0	0	0	0
	0	1	0	0	0	1
	0	0	0	0	0	0
	1	0	0	0	0	0
	0	1	0	0	0	1
	0	1	0	0	0	1
	0	0	0	0	0	0
	1	0	1	0	1	1
	0	1	0	0	0	1
	0	0	0	0	0	0
	0	0	0	0	0	0
	0					1
	0	1	0	0	0	1
	0	0	0	0	0	0
	0	0	0	0	0	0
	0	0	0	0	0	0
	0	0	0	0	0	0
	0	0	0	0	0	0
	1	0	0	0	0	0
	0	1	0	0	0	1
	0	0	0	0	0	0
	1	0	0	0	0	0
	0	0	0	0	0	0
	0	0	0	0	0	0
	0	0	0	0	0	0
	0	1	0	0	0	1
	0	0	0	0	0	0
	0	0	0	0	0	0
	0	0	0	0	0	0
	0	0	0	0	0	0
	0	0	0	0	0	0
	1	0	1	0	0	1
	0	0	0	0	0	0

0	0	0	0	0	0
0	0	0	0	0	0
1	0	0	0	0	0
1	1	0	0	0	1
0	0	0	0	0	0
1	0	0	0	0	0
0	0	0	0	0	0
0	0	0	0	0	0
0	0	0	0	0	0
0	0	0	0	0	0
0	0	0	0	0	0
0	0	1	0	1	1
0	0	0	0	0	0
0	0	0	0	0	0
0	0	0	0	0	0
0	0	0	0	0	0
0	0	0	0	0	0
0	0	0	0	0	0
0	1	0	0	0	1
0	0	0	0	0	0
0	0	1	0	1	1
1	0	0	0	0	0
0	0	0	0	0	0
1	1	0	0	0	1
1	1	0	0	0	1
1	0	1	0	1	1
0	0	0	0	0	0
1	1	0	0	0	1
1	1	0	0	0	1
1	0	0	1	1	1
1	0	0	1	1	1
0	0	0	1	1	1
1	0	0	1	1	1
1	0	0	1	1	1
1	0	0	1	1	1
1	0	0	1	1	1
